# Approaching the Dimerization Mechanism of Small Molecule Inhibitors Targeting PD-L1 with Molecular Simulation

**DOI:** 10.3390/ijms24021280

**Published:** 2023-01-09

**Authors:** Jianhuai Liang, Bingfeng Wang, Yang Yang, Boping Liu, Yulong Jin

**Affiliations:** Key Laboratory for Bio-Based Materials and Energy of Ministry of Education, College of Materials and Energy, South China Agricultural University, Guangzhou 510630, China

**Keywords:** PD-L1, small molecule inhibitor, dimerization mechanism, molecular dynamics simulation

## Abstract

Inhibitors blocking the PD-1/PD-L1 immune checkpoint demonstrate impressive anti-tumor immunity, and small molecule inhibitors disclosed by the Bristol-Myers Squibb (BMS) company have become a hot topic. In this work, by modifying the carbonyl group of BMS-202 into a hydroxyl group to achieve two enantiomers (MS and MR) with a chiral center, we found that this is an effective way to regulate its hydrophobicity and thus to reduce the negative effect of polar solvation free energy, which enhances the stability of PD-L1 dimer/inhibitor complexes. Moreover, we studied the binding modes of BMS-200 and BMS-202-related small molecule inhibitors by molecular dynamics simulation to explore their inhibitory mechanism targeting PD-L1 dimerization. The results showed that the size exclusion effect of the inhibitors triggered the rearrangement of the residue _A_Tyr56, leading to the formation of an axisymmetric tunnel-shaped pocket, which is an important structural basis for improving the binding affinity of symmetric inhibitors with PD-L1. Furthermore, after inhibitor dissociation, the conformation of _A_Tyr123 and _B_Met115 rearranged, which blocked the entrance of the binding pocket, while the reverse rearrangements of the same residues occurred when the PD-L1 monomer was complexed with the inhibitors, preparing PD-L1 for dimerization. Overall, this study casts a new light on the inhibitory mechanism of BMS inhibitors targeting PD-L1 dimerization and provides an idea for designing novel small molecule inhibitors for future cancer immunotherapy.

## 1. Introduction

Programmed cell death protein 1 (PD-1), an inhibitory receptor on activated T cells, can suppress the activity of activated T cells and maintain immune tolerance by binding to its ligand, namely programmed cell death 1 ligand 1 (PD-L1) [1,2,3,4]. However, tumor cells often evade attacks from the immune system by PD-L1 overexpression during tumor development. Inhibitors that rescue exhausted T cells by blocking the PD-1/PD-L1 interactions have demonstrated impressive anti-tumor immunity [5,6,7,8,9]. To date, more than a dozen monoclonal antibodies (mAbs) targeting PD-1 or PD-L1 (nivolumab, pembrolizumab, atezolizumab, etc.) have been approved for cancer treatment, and many mAbs are also in clinical trials [10,11,12,13,14,15]. Compared with mAbs, small molecule inhibitors are oral medicines with a shorter half-life and a lower molecular weight, which can avoid serious side effects and improve tissue penetrability and metabolic stability. Although at the early stage, the development of small molecule inhibitors targeting PD-L1 is promising [16,17,18,19].

**Figure 1:**
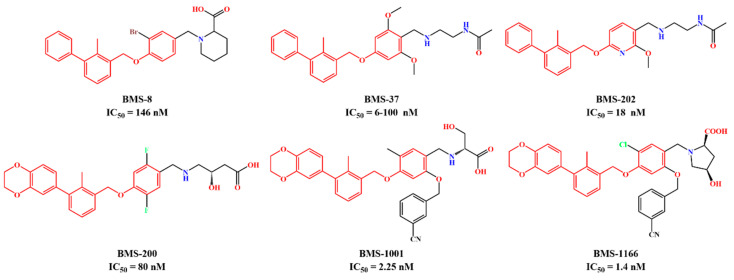
Two representative scaffolds of BMS small molecule inhibitors with IC_50_ values assayed by HTRF.

**Figure 2:**
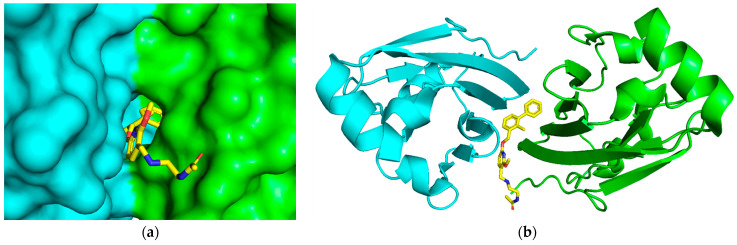
The binding pocket of the PD-L1 dimer/BMS-202 inhibitor crystal structure (PDB code: 5J89). BMS-202 was located at the interface of two PD-L1 chains, and the arrangement of the PD-L1 dimer is nearly axisymmetric. (**a**) Front view. (**b**) Top view. The native crystal conformations of BMS-202 are colored in yellow. Two monomers in PD-L1 dimer are annotated as _A_PD-L1 and _B_PD-L1 and colored in green and cyan, respectively.

The crystal structure of human PD-1/PD-L1 complex (PDB code: 4ZQK) resolved in 2015 provided precious structural information of the PD-1/PD-L1 interaction mode [20]. Meanwhile, a series of small molecule inhibitors blocking the PD-1/PD-L1 interaction by inducing PD-L1 dimerization were disclosed by the Bristol-Myers Squibb (BMS) company, and the half maximal inhibitory concentration (IC_50_) determined by homogeneous time-resolved fluorescence (HTRF) method ranged from the nM to µM level (Figure 1) [21,22]. Subsequently, the crystal structures of several BMS small molecule inhibitors bound to the PD-L1 dimer were resolved [23,24,25]. The inhibitors with the 2-methyl-3-(phenoxymethyl)-1,1′-biphenyl scaffold combined with the C’CFG strands of PD-L1 (the secondary structure identification is shown in Figure S1), which blocked the interface of the PD-1/PD-L1 interaction and induced PD-L1 dimerization to form the hydrophobic pocket (Figure 2). In addition, the structural rearrangement of the side chain of residue _A_Tyr56 was observed in the crystal structure analysis (the two monomers in the PD-L1 dimer were annotated by subscripts A and B), which turned the pocket into a hydrophobic tunnel [26]. This significant structural rearrangement coupled with the nearly axisymmetric character of the PD-L1 dimer inspired the idea of designing symmetric inhibitors to increase binding affinity [27,28,29]. Basu et al. modified the BMS-202 inhibitor to a symmetric inhibitor centered on a 2,2′-dimethyl-1,1′-biphenyl core, and the inhibitory activity of the symmetric inhibitor was nearly 3.8 times more potent than that of BMS-202 [30]. The results demonstrated that the symmetric small molecule inhibitors targeting the PD-L1 dimer were feasible.

Although the above experimental results provided the vital structural basis for a structure-based drug design, the mechanism of PD-L1 dimerization induced by BMS small molecule inhibitors is not well understood. Molecular simulation could be a unique way to approach the dimerization mechanism at a molecular level. Ahmed et al. investigated the conformational dynamics of the existing crystal PD-L1 structures by classical and accelerated molecular dynamics simulations, and the result of principal component analysis showed that the C’’D loop contributed to the maximum structural displacements and the conformation of PD-L1 was consistent with the conformational selection mechanism during the binding process [31]. Lim et al. analyzed the PD-L1/BMS small molecule inhibitor complexes with the fragment molecular orbital (FMO) method and determined residues of PD-L1: Tyr56, Glu58, Gln66, Met115, and Asp122 were key residues in hot spot regions [32]. Zhong et al. generated a pharmacophore model based on BMS small molecule inhibitors, which consisted of one hydrogen bond donor, three hydrophobic points, and one positive ionizable point, and identified residues of PD-L1: Ile54, Tyr56, Val68, Met115, and Ala121 were involved in generating hydrophobic interactions, and Asp122 and Lys124 were involved in forming hydrogen bonds [33]. Guo et al. investigated the inhibitory mechanism of PD-L1 dimer/BMS-200-related small molecule inhibitors by molecular dynamics simulation [34]. It was found that the inhibitors mainly bound to the key residues Ile54, Tyr56, Met115, Ala121, and Tyr123 on the PD-L1 dimer with non-polar interactions, and induced conformational changes in key residues. Moreover, the polar solvation free energy of the PD-L1 dimer/BMS-200 complex was highly unfavorable to binding affinity, which probably originated from the highly hydrophobic nature of the pocket. Likewise, Shi et al. assessed the binding free energies between PD-L1 and two BMS small molecule inhibitors (BMS-8 and BMS-1166) and obtained similar results [35].

Inspired by the above studies, in this work, we tentatively regulated the hydrophobicity of BMS-202 by modifying its carbonyl group into a hydroxyl group, which might weaken the negative effect of the polar solvation free energy, thus improving the stability of the PD-L1/inhibitor complexes [36]. It should be noted that this modification over BMS-202 generated a pair of enantiomers, which might exhibit a different potency towards PD-L1 dimerization. Therefore, it was mandatory to investigate both PD-L1 dimer/S-enantiomer and PD-L1 dimer/R-enantiomer complexes, named MS and MR systems, respectively. However, the following molecular-based studies showed that the chiral effect of the modified BMS-202 was negligible. For the sake of clarity, the results of both MS and MR systems will be given, but only the former will be discussed in detail.

Moreover, to investigate the inhibitory mechanism of BMS small molecule inhibitors, we comparably studied the binding mode of BMS-200 and BMS-202 inhibitors by molecular dynamics simulation. Moreover, three control groups were also established for comparison. Two of them were constructed by complexing S-enantiomer with the _A_PD-L1 and _B_PD-L1 monomer and marked as the SA and SB system, respectively, while the other was made only by the PD-L1 dimer, named the Dimer system (Table 1). The comprehensive analysis from the perspectives of energies, conformation, and binding modes on these systems can provide insight into the inhibitory mechanism of PD-L1 dimer/BMS small molecule inhibitors and help in identifying the key factors affecting the binding process, which might be useful for the design of novel small molecule inhibitors targeting PD-L1 dimerization as well.

## 2. Results and Discussion

### 2.1. Acquisition of the Initial Structure by Molecular Docking

**Figure 3:**
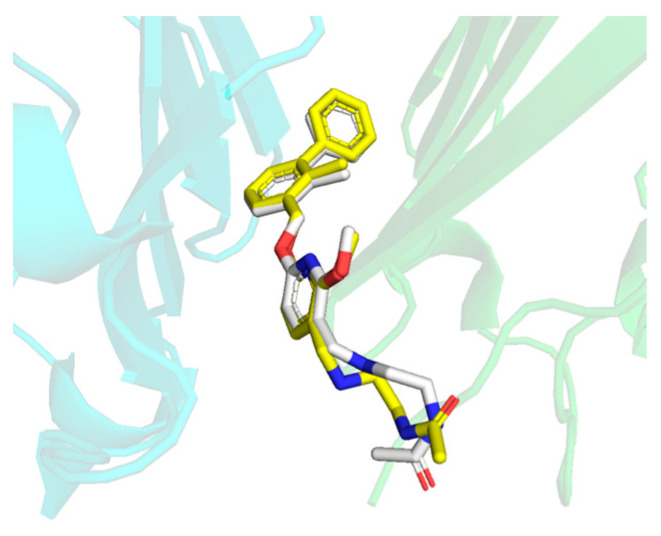
Superposition between the BMS-202 vina-docked conformation (yellow) and its native crystal conformation (white). Two chains in the PD-L1 dimer are annotated as _A_PD-L1 and _B_PD-L1 and colored in green and cyan, respectively.

The initial structures of the above systems were obtained by molecular docking and used for molecular dynamics (MD) simulations. In order to verify that the docking method was appropriate, the BMS-202 inhibitor was redocked to the PD-L1 dimer, and the conformation of the molecule in the docking and native crystal were almost superposed (Figure 3). The root mean square deviation (RMSD) was 1.507 Å, which verified that the docking method was appropriate. Therefore, the remaining small molecule inhibitors were docked to the PD-L1 dimer by the same docking method to obtain the initial structures.

### 2.2. Stability Evaluation of the Molecular Dynamics Simulation Results

**Figure 4:**
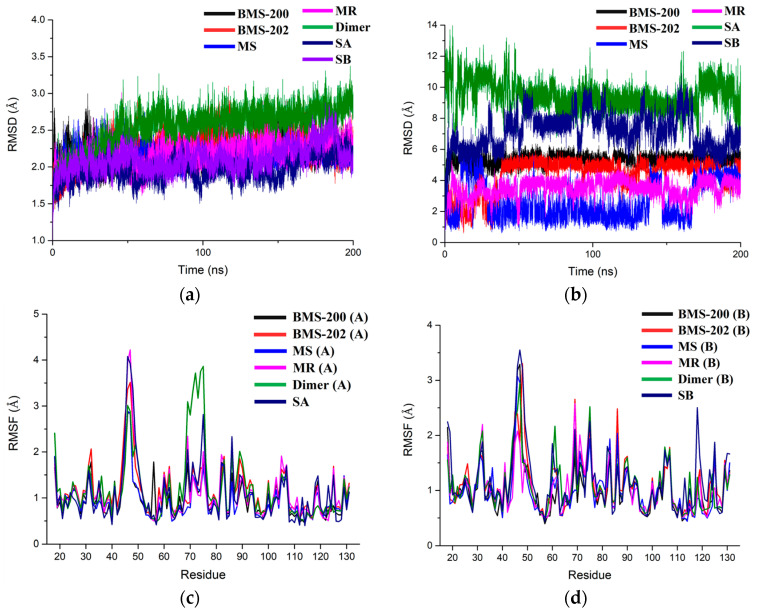
The RMSD and RMSF plots of all the systems during 200 ns MD simulation. (**a**) RMSD of protein heavy atoms. (**b**) RMSD of the inhibitors atoms. (**c**) RMSF of _A_PD-L1 residues. (**d**) RMSF of _B_PD-L1 residues.

The initial structures obtained by molecular docking were performed 200 ns MD simulations, and the RMSD of protein heavy atoms and inhibitors were used to assess whether the systems reached the equilibrium state (Figure 4 and Figure S2). As shown in Figure 4a, the deviation of protein heavy atoms in all the systems gradually increased in the first 50 ns simulations and then stabilized between 1.75 and 2.75 Å, which suggested that the protein structures had equilibrated and could be sampled for subsequent analysis. The Dimer system had the largest deviation, which indicated that the PD-L1 dimer became more flexible after inhibitor dissociation. As for the RMSD of the inhibitors (Figure 4b), the values for the two PD-L1 monomer/inhibitor systems were much higher than those for the PD-L1 dimer/inhibitor systems. Particularly, the SB system fluctuated significantly up and down, which implied that the inhibitors were less stable in binding with the PD-L1 monomer after losing the PD-L1 chain on either side. By observing the MD trajectories, it was found that the binding pocket formed by a single chain was too shallow to provide enough interaction, which led to inconstant conformation changes of the S-202 on the protein surface. In contrast, the RMSD curves were stabilized at a certain height in the PD-L1 dimer/inhibitor systems, and the rank of RMSD from highest to lowest among these systems was BMS-200, BMS-202, MR, and MS, which meant that, when bound with the PD-L1 dimer, the modified inhibitors were more stable than BMS-202.

In order to determine whether the protein of these systems had similar regional flexibility, root mean square fluctuation (RMSF) was calculated to evaluate the fluctuations of the protein structure referring to its average structure at each frame, and the results were divided into _A_PD-L1 and _B_PD-L1 for comparative analysis. As depicted in Figure 4c,d, the RMSF curves of _A_PD-L1 and _B_PD-L1 in all the systems basically overlapped, which suggested that the regional flexibility of PD-L1 in all systems was basically the same whether it was in the form of a dimer or monomer, with or without an inhibitor. Specifically, four dense peaks at 40–53 and 68–78 appeared in the residues of _A_PD-L1 and _B_PD-L1, respectively. According to the secondary structures of PD-L1, these peaks mainly appeared in the random coil regions with high flexibility, which were far away from the binding pocket and barely affected the binding process.

### 2.3. Comparative Analysis of Binding Free Energy

Considering the balance between computational cost and accuracy, the binding free energies of these systems were calculated by the MM-PBSA method to evaluate the binding affinity between PD-L1 and small molecule inhibitors (Table 2). With a regular gradient, the binding free energies of the four PD-L1 dimer/inhibitor systems in descending order were BMS-200, BMS-202, MR, and MS. Among these systems, BMS-200 bound to the PD-L1 dimer with less affinity than BMS-202, which was consistent with their inhibitory activities (see Figure 1). Modifying the carbonyl group of BMS-202 into a hydroxyl group can generally increase the binding affinity to the PD-L1 dimer. Moreover, the binding free energy between the two PD-L1 chains in the Dimer system was −32.38 kcal/mol, which implied that the PD-L1 dimer would not depolymerize in a short period after inhibitor dissociation. Two PD-L1 monomer/inhibitor systems, i.e., SA and SB, had much less binding affinity than the rest of the systems, which conformed with the RMSD results.

Specifically, the binding free energy (ΔG) was divided into polar and non-polar parts. Due to the hydrophobic surface of PD-L1, the non-polar binding free energy (Δ*G*_nonpl,total_) was favorable to the binding between PD-L1 and BMS small molecule inhibitors. Furthermore, the Δ*G*_nonpl,total_ can be divided into Van der Waals energy (Δ*E*_vdw_) and non-polar solvation free energy (Δ*E*_nonpl,sol_), where Van der Waals interaction was the main driving force during the binding process. In contrast, the polar binding free energy (Δ*G*_pol,total_) consisting of electrostatic energy (Δ*E*_ele_) and polar solvation free energy (Δ*E*_pol,sol_) was overall unfavorable to the binding process, mainly due to the serious negative effects of Δ*E*_pol,sol_.

The MS and MR systems had weaker electrostatic energy and polar solvation free energy than the BMS-202 system. It was distinct that, by simply modifying the carbonyl group of BMS-202 into a hydroxyl group, although the favorable electrostatic interactions became weaker, the value of polar solvation free energy was greatly reduced. Because the hydroxyl has weaker electronegativity than carbonyl, the polarity of the α-alkanol amino group consisting of the hydroxyl and the adjacent imino group was weaker than the amide group, and thus improved the hydrophobicity. Comparably, since BMS-200 was less hydrophobic than BMS-202, the BMS-200 system had much higher polar solvation free energy than that of the BMS-202 system, which became the main reason for the lower binding free energy of the latter system. By analyzing the binding free energy of the above systems, it was found that, although non-polar interactions were the driving force during the PD-L1/inhibitor binding process, decreasing the unfavorable polar interactions by balancing the hydrophilicity and hydrophobicity of the inhibitors was a more effective way to achieve high binding affinity.

A similar result can be found from the MM-PBSA data calculated by Shi et al., the electrostatic energies of BMS-8 and BMS-1166 were −29.29 and −72.23 kcal/mol, respectively; the polar solvation free energies of BMS-8 and BMS-1166 were 49.89 and 105.28 kcal/mol, respectively [35]. Comparing the structures of these two inhibitors (Figure 1), it can be seen that BMS-1166 was modified with additional structural fragments on the core scaffold, which increased the electrostatic interactions between the extra polar atoms and protein. It was found that, though the favorable electrostatic interaction was enhanced, the modification greatly improved the hydrophilicity of BMS-1166 and significantly increased the unfavorable polar solvation free energy, which was consistent with the conclusion drawn in this work.

### 2.4. Key Residue Recognition

**Figure 5:**
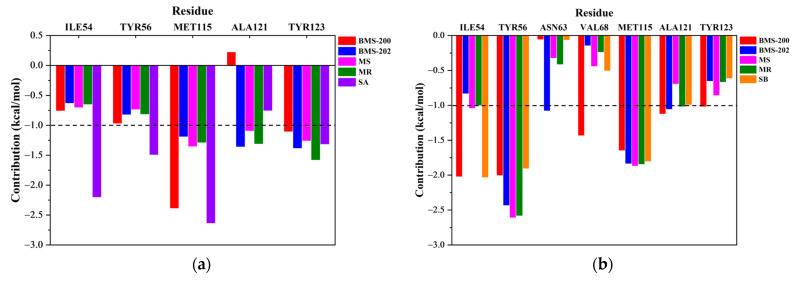
The energy contribution of the residues from (**a**) _A_PD-L1 and (**b**) _B_PD-L1 in all systems (all residues identified to be key residues in any systems are shown).

**Figure 6:**
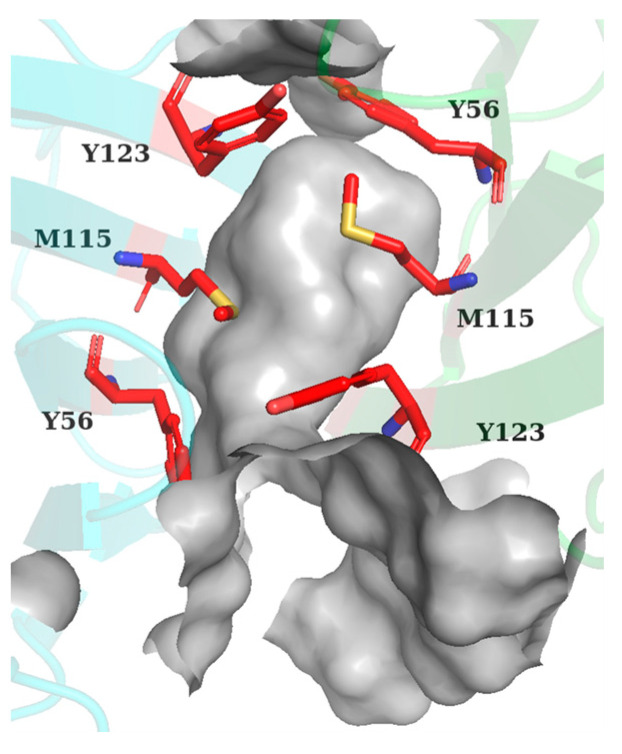
Spatial residue distribution of BMS-202 system. The distribution of key residues (red) was almost axisymmetric (similar distributions of these residues could also be found in other PD-L1 dimer/inhibitor systems). _A_PD-L1 and _B_PD-L1 are colored in green and cyan, respectively. Top view of the binding pocket, and the surface of pocket are colored in gray.

The binding free energies obtained above were decomposed and attributed to each residue of PD-L1, in which the residues with an energy contribution lower than −1 kcal/mol were defined as key residues. As illustrated in Figure 5, _A_Met115, _A_Tyr123, _B_Tyr56, and _B_Met115 were commonly identified as key residues for all systems. In addition, residues such as _A_Ile54, _A_Tyr56, _A_Ala121, _B_Ile54, _B_Ala121, and _B_Tyr123 were found to make significant contributions to different systems as well, which indicated that the binding mode of these systems were similar, and the identities of the important residues were almost the same in both monomers of the PD-L1 dimer. Moreover, the spatial distribution of these important residues showed that they were almost axisymmetrically located in the pocket (Figure 6). Altogether, it laid the structural basis for designing small molecule inhibitors with geometrical symmetry, which have been experimentally verified to exhibit better efficacy [30].

**Figure 7:**
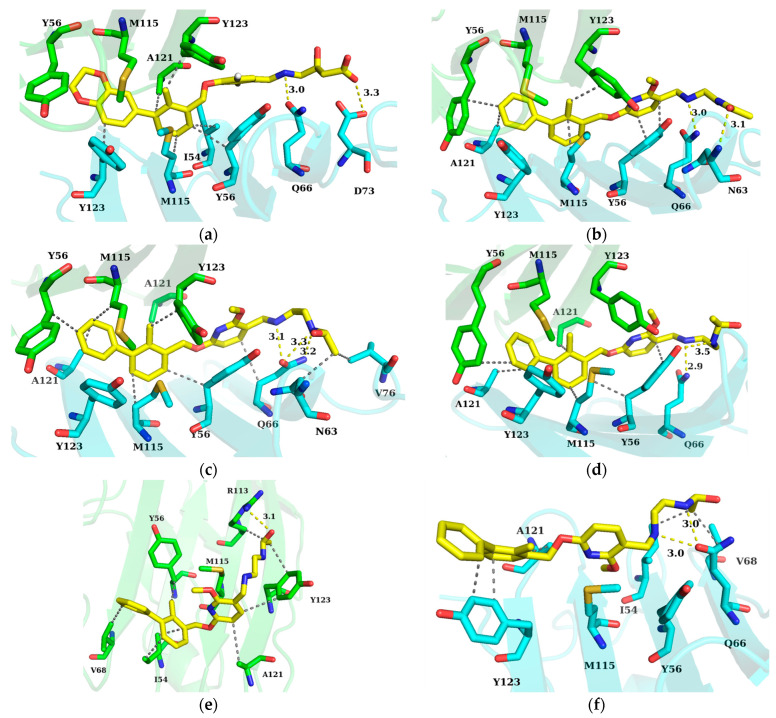
Interaction pattern of each system. (**a**) BMS-200 system. (**b**) BMS-202 system. (**c**) MS system. (**d**) MR system. (**e**) SA system. (**f**) SB system. The _A_PD-L1 and _B_PD-L1 are colored in green and cyan, respectively, while small molecule inhibitors are colored in yellow. Hydrogen bonds and non-polar interaction are shown as yellow and gray dotted lines, respectively.

However, differences could also be found after detailed comparisons. For example, with respect to the residues which contributed most significantly to the binding, it was _A_Met115 for the BMS-200 system, while it changed to _B_Tyr56 for the others. Similarly, _A_Ala121 made negative contributions to the BMS-200 system, but it was a key residue in the other PD-L1 dimer/inhibitor systems. Moreover, due to the slight displacement of the binding site for SA system, the key residues of SA system were different from BMS-202 system (Figure 7).

### 2.5. Hydrogen Bond Analysis

In order to explore the mechanism during the binding process, hydrogen bond statistics were used to analyze the hydrogen bond occupancy between the inhibitors and PD-L1 of the last 50 ns trajectories. The number of hydrogen bonds of each system during the whole MD simulation is shown in Figure S2. All systems had no more than five hydrogen bonds in each frame, which conformed to the Rule of Five [37,38]. The number of hydrogen bonds was highest in the BMS-200 and BMS-202 systems, followed by the MS and MR systems and then the SA and SB systems, which was consistent with the magnitude of electrostatic energy contributed by hydrogen bond interactions. The occupancies of hydrogen bonds are listed in Table 3.

It is worth noting that all hydrogen bonds with high occupancy were formed between _B_PD-L1 and the linear tail of the inhibitors in the four PD-L1 dimer/inhibitor systems. These hydrogen bonds mainly formed at the outlet of the binding pocket, where the residues such as His, Asp, and Glu belonging to _B_PD-L1 were charged, while residues such as Ala, Phe, and Thr belonging to _A_PD-L1 were uncharged. Moreover, the flexible linear tail of the inhibitors contained high electronegative atoms such as nitrogen and oxygen, which were easily attracted to the surface of _B_PD-L1 by electrostatic interaction to form hydrogen bonds with the charged residues.

### 2.6. Binding Mode Analysis and Residue Rearrangement Investigation

Subsequently, the non-bonded interactions between the inhibitors and PD-L1 were evaluated. Based on the results of interaction analysis and key residue recognition, we had a full view of the interaction pattern of each system. As illustrated in Figure 7, for the PD-L1 dimer/inhibitor systems, the biphenyl ring of the inhibitors was inside the binding pocket, while the pyridine ring and tail chain of the inhibitors were exposed to solvent at the outside of the binding pocket. With the hydrophobic environment formed by the narrow and deep binding pocket, numerous non-polar interactions were formed between the phenyl ring, pyridine ring, and surrounding residues. The staggered phenyl rings occupied a large space, which allowed the inhibitors to be tightly stuck in the binding pocket. In addition, the linear flexible tail of the inhibitors coupled with hydrogen bond interactions provided a good match to the uneven surface of the binding pocket.

**Figure 8:**
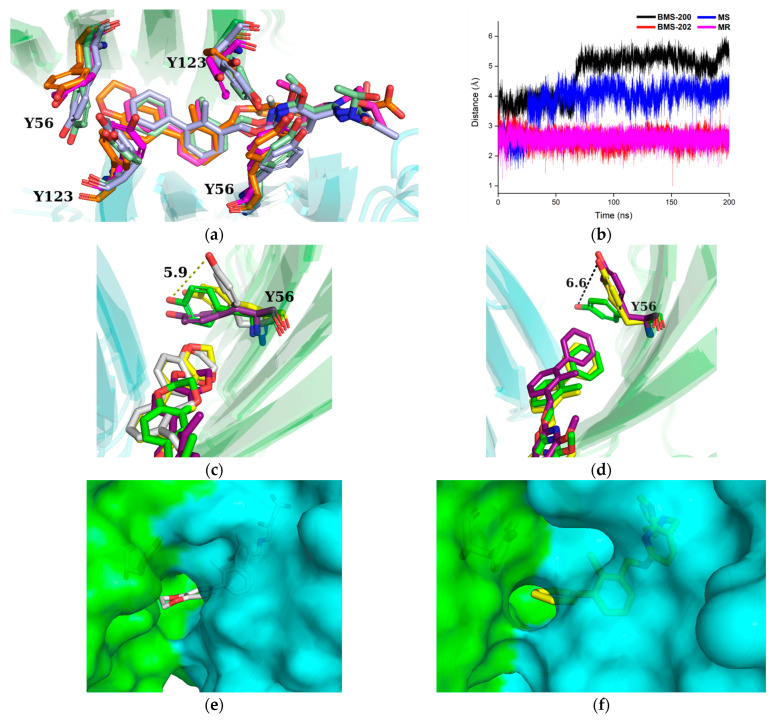
Conformational changes of the BMS-200 and MS systems during MD simulations. The side chain of residue _A_Tyr56 exhibited significant conformational rearrangement in the BMS-200 and MS systems. The hole formed at the bottom of the binding pocket turned the unilateral opening pocket into a through tunnel. (**a**) Conformational analysis by PD-L1 structural alignment. The BMS-200, BMS-202, MS, and MR systems are colored in orange, light blue, magenta, and pale green, respectively. (**b**) Minimum distance between the inhibitors and the geometric center of the side chain of _A_Tyr56. The curves were smoothed to remove irregular noisy data by the adjacent-averaging filter with five points of window. Motional visualization of the BMS-200 system (**c**) and MS system (**d**). The structure of each frame is colored in green, purple, yellow, and white, in chronological order. The distance between the two frames with the largest conformational difference is represented by the yellow dotted line. The conformational changes of the binding pocket (rear view) in the BMS-200 system (**e**) and MS system (**f**). The _A_PD-L1 and _B_PD-L1 are colored in green and cyan, respectively.

Furthermore, the structures of four PD-L1 dimer/inhibitor systems were aligned by the PD-L1 dimer (Figure 8a). It can be seen that the conformations of the inhibitors were very similar. Among the four Tyr residues in the binding pocket, only _A_Tyr56 clearly exhibited conformational rearrangement, which was in line with the results of crystal analysis [23].

In order to provide insight on the mechanism behind the rearrangement of _A_Tyr56, the minimum distances between the inhibitors and the geometric center of the side chain of _A_Tyr56 are shown in Figure 8b. The minimum distances of the BMS-202 and MR systems remained broadly the same. In contrast, for the BMS-200 and MS systems, the minimum distance increased abruptly and remained stable. According to the trajectories describing the displacement between _A_Tyr56 and the inhibitors (see Figure 8c,d), BMS-200 moved forward to _A_Tyr56, causing the side chain of _A_Tyr56 to twist away from the inhibitor and flip upward, which opened an additional hole at the bottom of the binding pocket and turned the unilateral opening pocket into a through tunnel. By contrast, BMS-202 was still deeply embedded in the binding pocket. Moreover, the hole formed in the BMS-200 system was larger than that of the MS system because the BMS-200 molecule has an additional 1,4-dioxane fragment at the head of its structure.

In summary, both a T-stacking interaction and a size exclusion effect existed between the inhibitors and _A_Tyr56. Since the motion of inhibitors was more intense than that of _A_Tyr56, the inhibitors tended to stagger with the phenyl plane of _A_Tyr56, weakening the T-stacking interaction. Moreover, the size exclusion effect between the inhibitors and the side chain of _A_Tyr56 would separate them further, causing the rearrangement of the side chain of _A_Tyr56.

### 2.7. Correlation Analysis of Residues Motion

**Figure 9:**
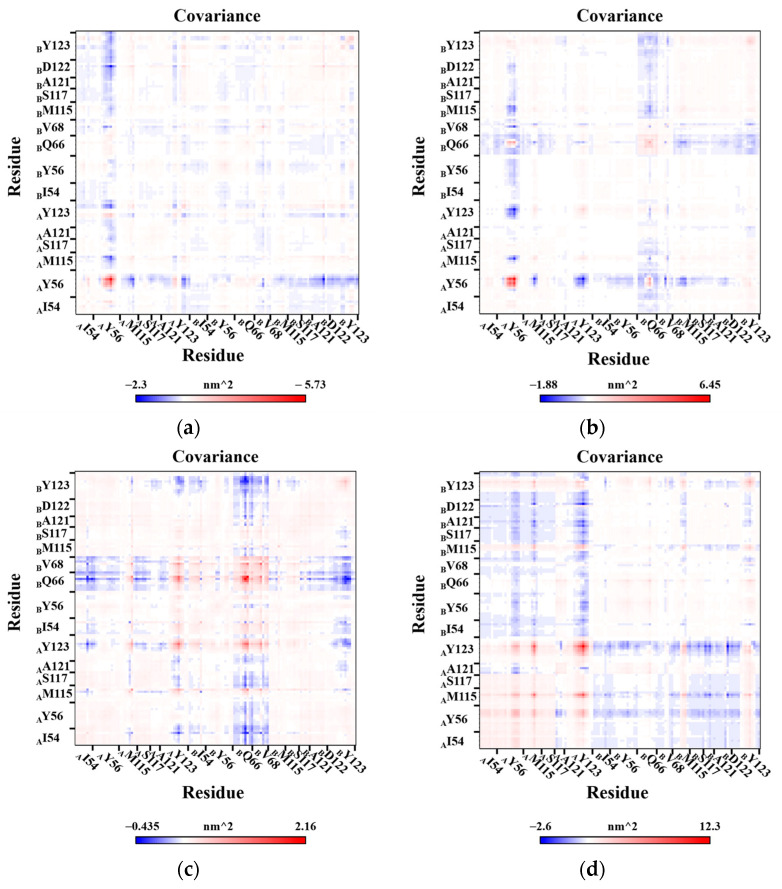
Cross-correlation matrices for the heavy atoms of residues in the binding pocket. (**a**) BMS-200 system. (**b**) MS system. (**c**) MR system. (**d**) Dimer system. The anti-correlated, uncorrelated, and correlated motions among the residues are colored in blue, white, and red, respectively. The color depth on the diagonal of the matrix reflected the motion intensity of the residue itself.

Furthermore, to better investigate the motion behaviors of the residues in the binding pocket, the cross-correlation matrix was used to analyze the direction, amplitude, and motion correlation of the residues in different systems (Figure 9). For the BMS-200 system, the motion of _A_Tyr56 was very active, which was anti-correlated with the other residues. This anti-correlation was more obvious between _A_Tyr56 and the nearby residues _B_Asp122 and _B_Tyr123. As expected, a similar situation occurred in the MS system, but there were evident anti-correlated motions between residue _A_Tyr56 and _A_Tyr123 rather than _B_Tyr123. In addition, _B_Gln66 also had anti-correlated motions with _B_Tyr123 and _A_Tyr56 in the MS system. For MR system, similar motion behavior was not observed in _A_Tyr56, which conformed with the previous results that only _A_Tyr56 in the BMS-200 and MS systems showed notable conformational changes. Moreover, the anti-correlated motions between _B_Gln66 and both _A_Ile54 and _B_Tyr123 were more intense, and there were synergetic motions between _B_Gln66 and the adjacent residues in the MR system.

For the Dimer system, correlated motions between the residues of _A_PD-L1 were observed clearly, while general anti-correlated motions between the residues of _A_PD-L1 and those of _B_PD-L1 were also evident, which indicated that the overall motional directions of the two PD-L1 chains were opposing. Therefore, the PD-L1 dimer showed a separation tendency after inhibitor dissociation.

### 2.8. Free Energy Landscapes and New Insight into PD-L1 Dimerization

**Figure 10:**
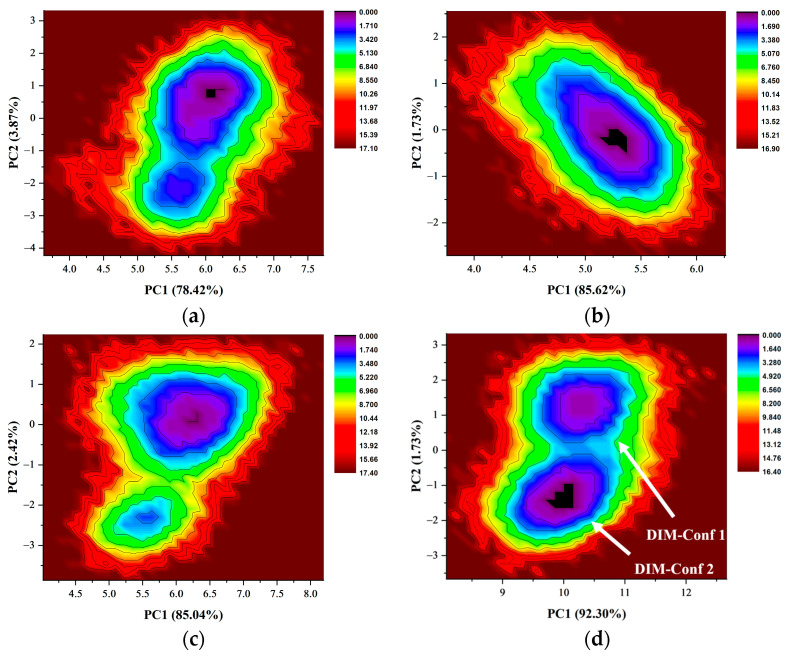
Free energy landscapes of the binding pocket in (**a**) the BMS-200 system, (**b**) the BMS-202 system, (**c**) the MS system, and (**d**) the Dimer system. The free energy landscapes of the residues in the binding pocket were generated by projecting the last 50 ns trajectories onto the two largest principal components (PCs), which were obtained from the diagonalization of the covariance matrices in Figure 9. The two metastable clusters of the Dimer system are named DIM-Conf 1 and 2, respectively. The conformation of these residues could potentially switch between the two clusters. The number in parentheses shows the percentage of the variance of PCs in the total variance. The energy of the conformation is represented by the color legend.

**Figure 11:**
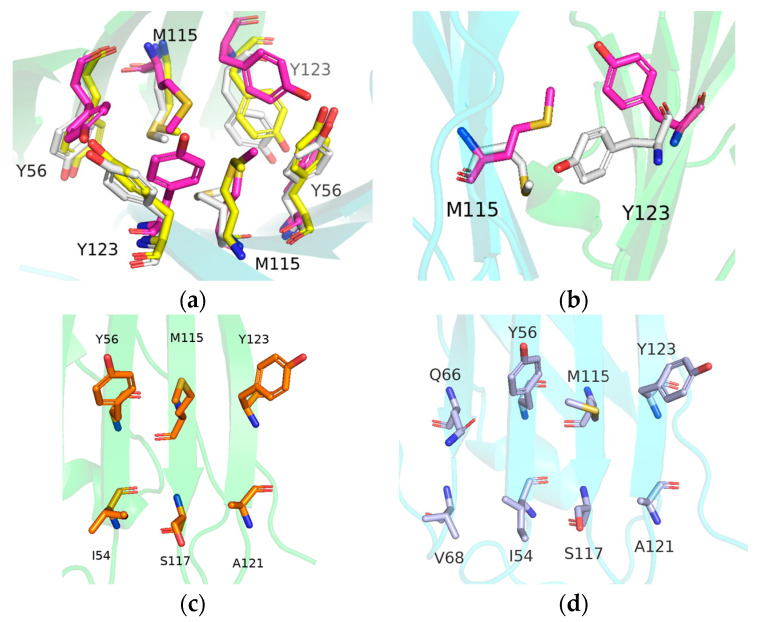
(**a**) Conformational differences among the stable conformation of the MS and Dimer system. (**b**) The entrance of the binding pocket was completely blocked by the conformational changes of residues _A_Tyr123 and _B_Met115, resulting in pocket disappearance. In the SA (**c**) and SB (**d**) systems, PD-L1 combined with the inhibitor to form an open conformation in preparation for PD-L1 dimerization. MS, SA, SB, DIM-Conf 1, and DIM-Conf 2 conformation are colored in magenta, orange, light blue, yellow, and white, respectively. _A_PD-L1 and _B_PD-L1 are colored in green and cyan, respectively.

Subsequently, the above covariance matrices were diagonalized to obtain principal components (PCs), and the last 50 ns trajectories were then projected onto the two largest principal components (PC1 and PC2) to characterize the free energy landscapes of the binding pocket. The results of all systems are shown in Figure 10 and S3. The consecutive conformational spaces of the four PD-L1 dimer/inhibitor systems were mainly located in the range of 4 < PC1 < 7 and −3 < PC2 < 3, which indicated that the four PD-L1 dimer/inhibitor systems had converged to the same space. However, the distribution of low-energy regions of each system has its own characteristics. For the BMS-202 and MS systems, a large and unique low-energy region demonstrated that residues in the binding pocket tended to maintain a stable conformation. However, there were two low-energy regions in the BMS-200, MR, and Dimer systems, which implied that their residues could potentially switch between the two conformation. The two conformations in the Dimer system, named DIM-Conf 1 and 2, respectively, were compared with the low-energy conformation of the MS system (Figure 11).

It was found that the side chains of the four Tyr residues were on the same side in the MS system. For the DIM-Conf 1 and 2 conformations, due to inhibitor dissociation, Tyr56 and Tyr123 have extra space to stagger up and down, which weakened the size exclusion effect and led to a new stable conformation. In addition, the side chains of residue _B_Met115 and _A_Tyr123 in DIM-Conf 1 and 2 showed a downward flip, leading to the reduction of the cavity of the binding pocket. As a consequence, the entrance of the binding pocket was completely blocked. On the contrary, when a PD-L1 monomer was complexed by the inhibitors, the PD-L1 monomer would undergo reverse rearrangement and prepare for dimerization with nearby PD-L1.

## 3. Materials and Methods

### 3.1. Molecular Modeling

The PD-L1/BMS-200 and PD-L1/BMS-202 crystal complexes (PDB codes: 5N2F, 5J89) were downloaded from the Research Collaboratory for Structural Bioinformatics (RCSB) Protein Data Bank [23,26,39]. The initial structures of BMS-200 and BMS-202 were extracted from the crystal complexes. The S and R enantiomers were obtained by modifying the carbonyl group of BMS-202 into a hydroxyl group. Structural optimization of all the small molecule inhibitors was carried out at the B3LYP/def2SVP level with the Gaussian09 E.01 package, and RESP atomic charges were calculated with Multiwfn 3.7 [40,41]. The missing sidechains of protein structures were repaired through the WHAT IF online server.

### 3.2. Molecular Docking

Molecular docking is a technique of placing an inhibitor into the binding pocket to obtain a series of matching patterns of interaction. Autodock Vina was used for docking small molecule inhibitors into the PD-L1 dimer [42]. We built a 22 × 18 × 18 grid box with 1 Å grid spacing centering on inhibitors, and the energy range and exhaustiveness were set as 4 kcal/mol and 24, respectively [43,44]. According to the affinity evaluation by scoring function and binding mode analysis, the best mode was selected as the initial structure for molecular dynamics simulation [45,46].

### 3.3. Molecular Dynamics Simulation

Molecular dynamics simulation is a powerful tool for analyzing the conformational change of protein and the kinetic characteristics of small molecule inhibitors at an atomic level. For all the systems above, the MD simulations were carried out using the Gromacs 2016 software package [47]. The GAFF and AMBER 14SB force fields were used to characterize the small molecule inhibitors and PD-L1 protein structures, respectively [48,49]. The systems were solvated in a periodic cubic box filled with TIP3P water molecules and electrically neutralized with counterions. The minimum distance between the protein and the edge of the box was set as 10 Å to remove the boundary effect. In order to relax close contacts in the starting structures, the steepest decent (SD) and conjugated gradient (CG) algorithms were employed to minimize the maximum force of the systems to less than 50 kJ/mol·nm. Next, the systems were heated up from 0 to 300 K in 1 ns NVT equilibration using a V-rescale thermostat with all protein and inhibitor atoms fixed. The systems were given an initial velocity consistent with the Maxwell distribution. After that, the pressure of the systems was coupled at 1 bar in 1 ns NPT equilibration using the Berendsen algorithm with all protein and inhibitor atoms fixed. Finally, 200 ns production simulations were performed without any positional constraints, and snapshots of the last 50 ns trajectory were taken every 1.0 ps for subsequent analyses. During the production simulations, the LINCS algorithm was used to constrain the hydrogen bonds, the Particle Mesh Ewald (PME) was used for the long-range electrostatics calculation, the cut-off method was used for the short-range van der Waals force calculation and short-range electrostatics calculation (the cut-off values were set as 1 nm), the V-rescale was used for temperature coupling, and Parrinello–Rahman was used for pressure coupling. The remaining parameters were set to default values. All simulations were repeated with the same parameters five times to ensure stability and repeatability.

### 3.4. Binding Free Energy

For the MD trajectory of biomolecular systems, the MM-PBSA method is commonly used to calculate the protein–inhibitor binding free energy (ΔG) to determine the binding affinity between protein and inhibitor [50,51]. In this work, 500 snapshots extracted from the last 50 ns trajectory of the MD simulation were used to calculate the binding free energy by the g_mmpbsa tool [52]. The simplified equations for calculating the binding free energy are shown below:

ΔG = G_com_ − G_pro_ − G_lig_ (1)

G = E_MM_ − TΔS + G_sol_ (2)

E_MM_ = E_vdw_ + E_ele_ (3)

G_sol_ = E_pol,sol_ + E_nonpl,sol_ (4)

E_nonpl,sol_ = γ·ΔSASA (5)

where G_com_, G_pro_, and G_lig_ are the free energy of the PD-L1/inhibitor complex, PD-L1 dimer, and small molecule inhibitors, respectively. The energy (E_MM_) calculated by molecular mechanics consists of the energy of van der Waals (E_vdw_) and electrostatic interaction (E_ele_). The solvation free energy (G_sol_) is comprised of the polar solvation free energy (E_pol,sol_) and non-polar solvation free energy (E_nonpl,sol_). ΔSASA denotes the solvent-accessible surface areas, and all the empirical parameters, including γ, are set to default. Considering that the entropy contribution (ΔS) is computationally expensive and tends to have a large margin of error that introduces significant uncertainty to the results, the ΔS of all systems were ignored [53,54]. In addition, all energy terms were subdivided and attributed to each residue to identify the key residues for the binding process.

## 4. Conclusions

In this work, to explore the effect of the hydrophobicity of the inhibitors on its binding affinity, we modified the carbonyl group of BMS-202 into a hydroxyl group, which generated a pair of enantiomers (MS and MR) and was complexed with the PD-L1 dimer. Meanwhile, we compared the binding modes of BMS-200, and these BMS-202-related small molecule inhibitors by molecular dynamics simulation to investigate their inhibitory mechanism targeting PD-L1 dimerization. Moreover, three control groups were also established for comparison. Two of them were constructed by complexing the S-enantiomer (MS) with the _A_PD-L1 and _B_PD-L1 monomer, respectively, while the other was made only by the PD-L1 dimer.

It was found that the chemical modification could effectively improve the hydrophobicity of BMS-202 and greatly reduced the negative effect of polar solvation energy, which enhanced the stability of the PD-L1 dimer/inhibitor complexes, providing an idea for the structural modification of small molecule inhibitors. Regarding the inhibitory mechanism of BMS inhibitors targeting PD-L1, it can be concluded that the staggered phenyl rings were the key fragment of the inhibitors, which occupied a large space and formed numerous non-polar interactions with surrounding residues, allowing the inhibitors to be firmly anchored in the pocket. Complementally, the linear flexible tail of the inhibitors caused hydrogen bond interactions to match the uneven surface of the binding pocket. Moreover, both a T-stacking interaction and a size exclusion effect existed between the inhibitors and _A_Tyr56, and the size exclusion effect triggered the rearrangement of the side chain of _A_Tyr56, resulting in the formation of an axisymmetric tunnel-shaped pocket, which is an important structural basis for improving the binding affinity of symmetric inhibitors. Moreover, the downward conformational rearrangements of residues _A_Tyr123 and _B_Met115 completely blocked the entrance of the binding pocket after inhibitor dissociation, such that other molecules could not access it, while the reverse rearrangement occurred when the PD-L1 monomer was complexed with the inhibitors, elucidating the key mechanism of PD-L1 dimerization.

Overall, this study casts a new light on the inhibitory mechanism of BMS small molecule inhibitors targeting PD-L1 dimerization and provides an idea for the design of novel small molecule inhibitors.

## Figures and Tables

**Table 1:** An overview of the investigated systems in this work.

System	Receptor	Inhibitor	Structure of Inhibitor
BMS-200	PD-L1 Dimer	BMS-200	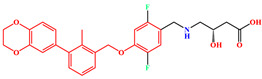
BMS-202	PD-L1 Dimer	BMS-202	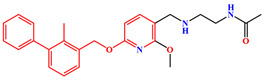
MR	PD-L1 Dimer	R-202	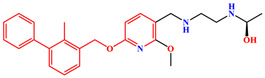
MS	PD-L1 Dimer	S-202	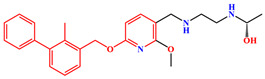
SA	_A_PD-L1		
SB	_B_PD-L1		
Dimer	PD-L1 Dimer	/	/

**Table 2:** The binding free energies of the systems evaluated by the MM-PBSA method.

Contribution ^a^	BMS-200	BMS-202	MS	MR	Dimer ^i^	SA	SB
Δ*E*_vdw_^b^	−66.45 ± 0.15	−64.58 ± 0.13	−62.57 ± 0.17	−63.30 ± 0.12	−50.76 ± 0.29	−34.17 ± 0.17	−32.32 ± 0.16
Δ*E*_ele_^c^	−10.93 ± 0.23	−7.82 ± 0.09	−4.28 ± 0.09	−4.18 ± 0.09	−168.16 ± 1.03	−4.84 ± 0.17	−2.31 ± 0.09
Δ*E*_pol,sol_^d^	40.83 ± 0.29	34.47 ± 0.16	26.38 ± 0.14	28.38 ± 0.12	193.76 ± 1.32	14.68 ± 0.28	12.46 ± 0.18
Δ*E*_nonpl,sol_^e^	−5.77 ± 0.01	−5.23 ± 0.01	−5.22 ± 0.01	−5.02 ± 0.01	−7.23 ± 0.03	−3.62 ± 0.02	−3.53 ± 0.01
Δ*G*_pol,total_^f^	29.91 ± 0.52	26.65 ± 0.26	22.10 ± 0.24	24.20 ± 0.21	25.60 ± 2.35	9.83 ± 0.45	10.14 ± 0.27
Δ*G*_nonpl,total_^g^	−72.22 ± 0.16	−69.82 ± 0.14	−67.80 ± 0.19	−68.32 ± 0.13	−57.99 ± 0.32	−37.80 ± 0.19	−35.84 ± 0.17
Δ*G* ^h^	−42.32 ± 0.16	−43.17 ± 0.13	−45.70 ± 0.17	−44.13 ± 0.13	−32.38 ± 0.53	−27.96 ± 0.16	−25.71 ± 0.17

^a^ All the energy contributions are in kcal/mol. ^b^ Van der Waals energy. ^c^ Electrostatic energy. ^d^ Polar solvation free energy. ^e^ Non-polar solvation free energy. ^f^ Total polar binding free energy (consisting of electrostatic energy and polar solvation free energy). ^g^ Total non-polar binding free energy (consisting of Van der Waals energy and non-polar solvation free energy). ^h^ Binding free energy. ^i^ The value of the Dimer system is the binding free energy between two PD-L1 chains.

**Table 3:** Hydrogen bond occupancy of the systems.

System	Donor	Receptor	Occupancy (%) ^a^
BMS-200	BMS-200@O3	_B_Asp73@OD1	56.67 ± 1.88
	_B_His69@NE2	BMS-200@O2	19.71 ± 2.41
	BMS-200@O1	_B_Asp73@OD2	15.16 ± 2.17
BMS-202	_B_Glu66@NE2	BMS-202@N1	80.55 ± 1.57
	_B_Asn63@N	BMS-202@O2	73.75 ± 1.98
MS	S-202@N2	_B_Gln66@OE1	48.47 ± 2.72
MR	_B_Gln66@NE2	R-202@N2	60.79 ± 1.58
	R-202@N1	_B_Tyr56@OH	17.15 ± 2.15
SA	Arg113@NE	S-202@O2	12.96 ± 2.74
	Arg113@NH2	S-202@N1	11.36 ± 2.16
	Arg113@NH1	S-202@O2	11 ± 2.02
SB	S-202@N2	Gln66@OE1	27.69 ± 3.26

^a^ Only hydrogen bonds with a Donor–Acceptor atom distance less than 3.5 Å, a Donor–H acceptor angle greater than 120°, and an occupancy greater than 10% are listed.

## Data Availability

The data presented in this study are available within the article, figures, tables, and supplementary materials.

## Supplementary Materials

The supporting information can be downloaded at https://www.mdpi.com/article/10.3390/ijms24021280/s1.
