# Supplementary material for: Approaching the Dimerization Mechanism of Small Molecule Inhibitors Targeting PD-L1 with Molecular Simulation
Source: Int J Mol Sci. 2023 Jan 9;24(2):1280. doi: 10.3390/ijms24021280 (PMC9866166; doi:10.3390/ijms24021280)
Supplement: Supplementary file 1 [file ijms-24-01280-s001.zip › ijms-2100504-supplementary.pdf]

Approaching the Dimerization Mechanism of Small Molecule Inhibitors Targeting PD-L1 with Molecular Simulation (Supporting Information)

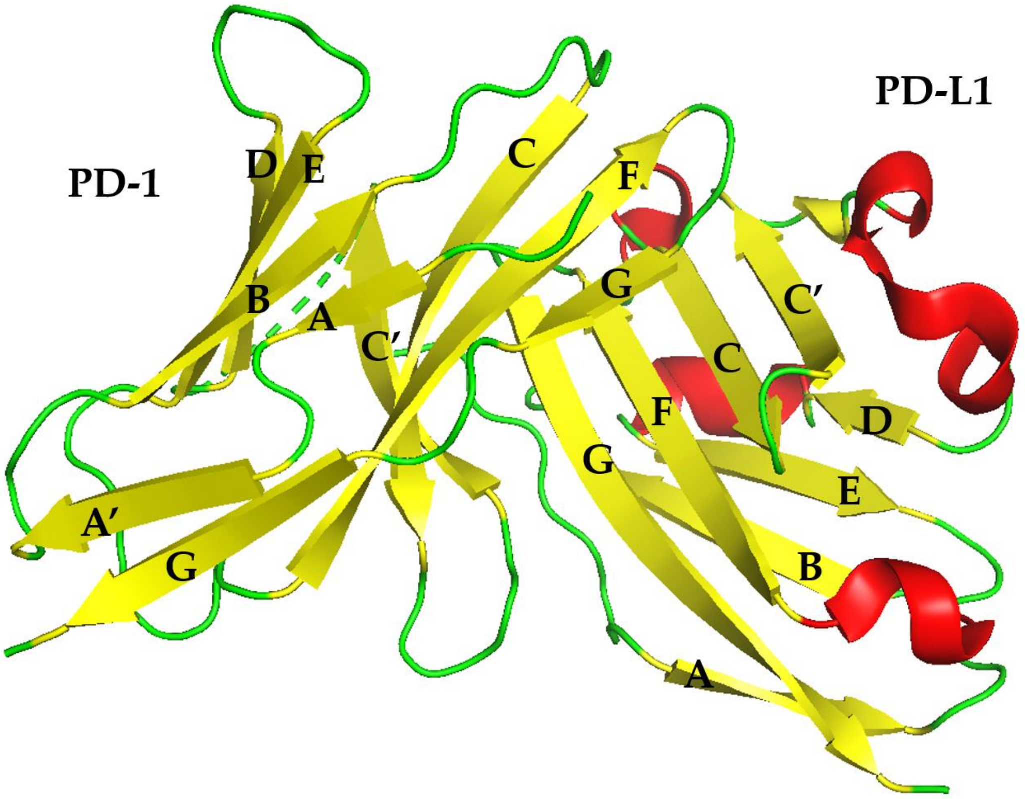

Figure S1. The crystal structure of human PD-1/PD-L1 complex in cartoon mode (PDB code: 4ZQK). The secondary structures of helix, β sheet and random coil region were colored with red, yellow and green, respectively.

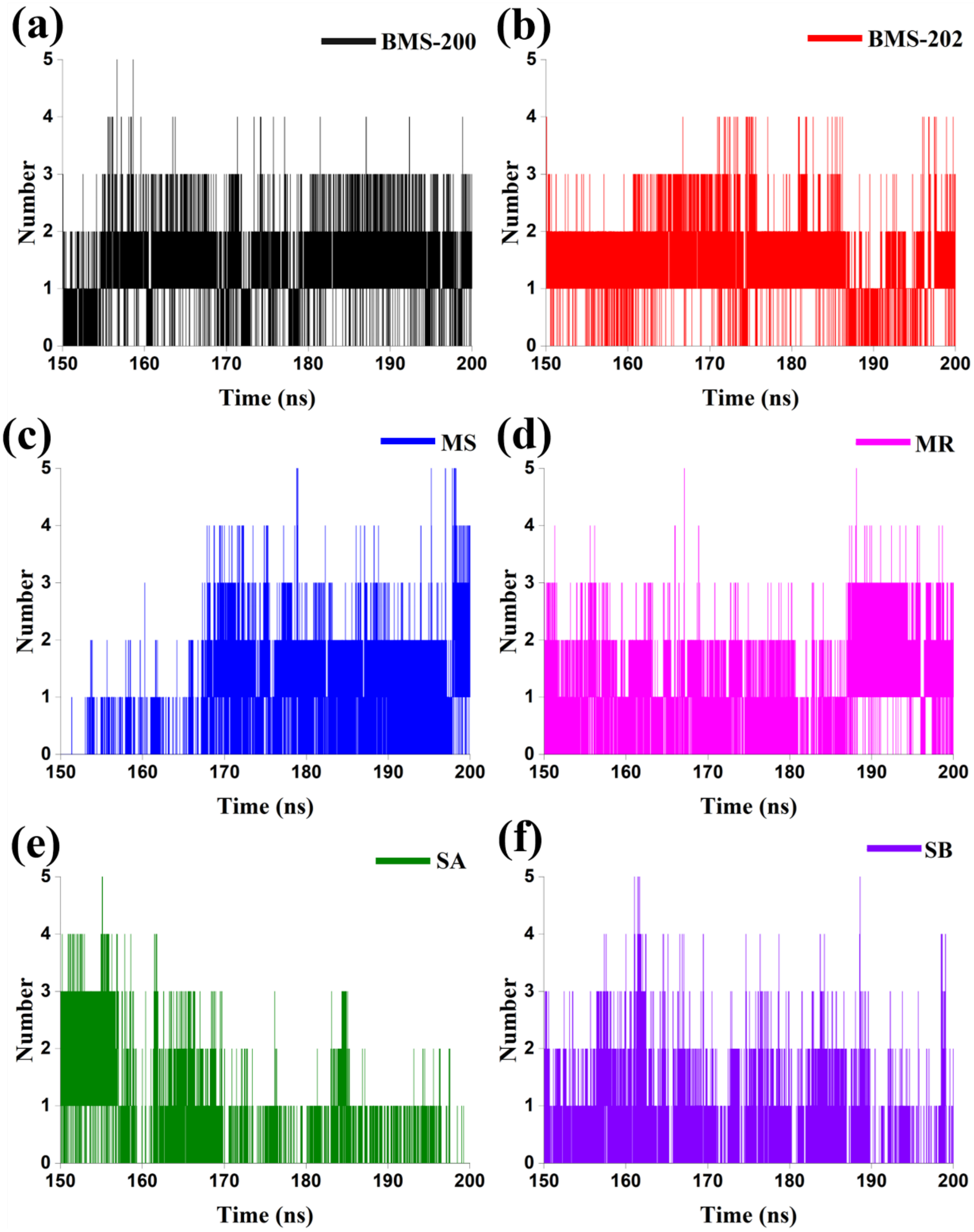

Figure S2. Number of hydrogen bonds of all the systems during MD simulation. (a) BMS-200 system. (b) BMS-202 system. (c) MS system. (d) MR system. (e) SA system. (f) SB system.

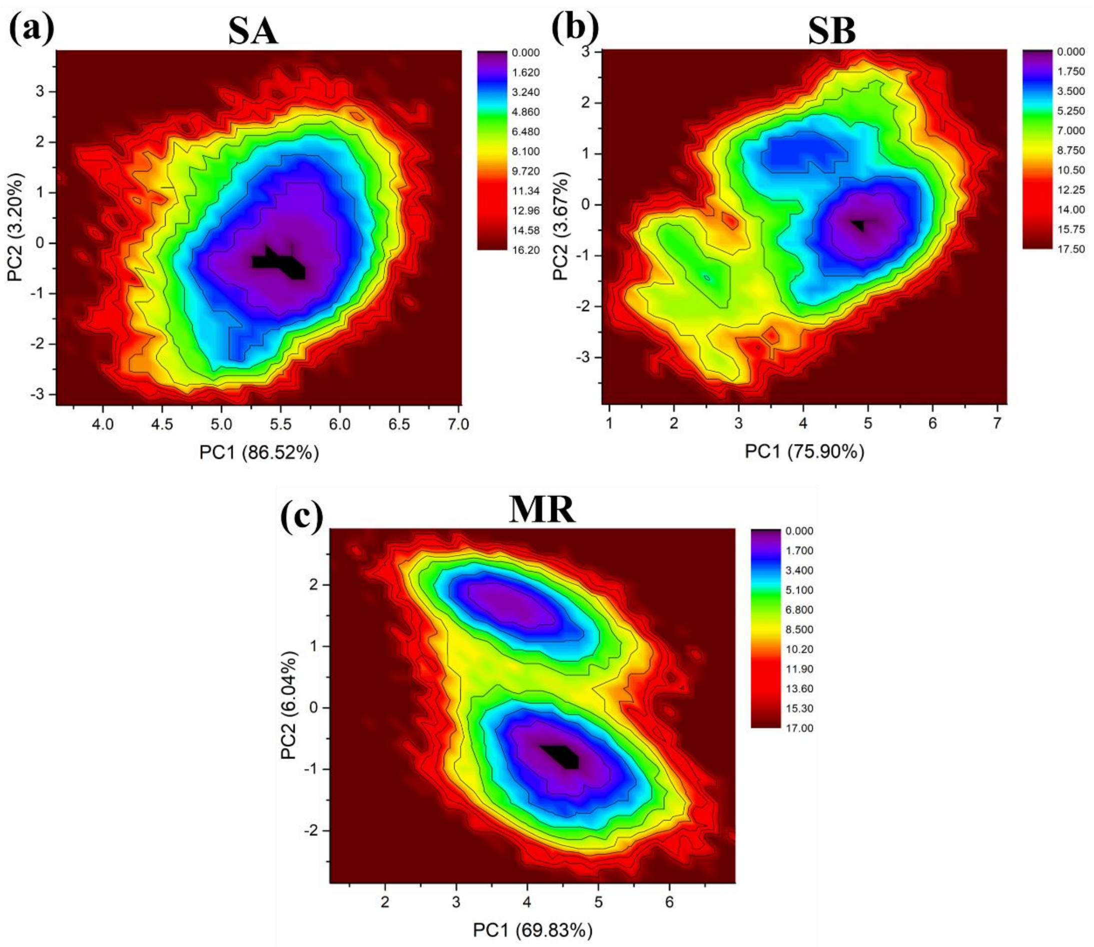

Figure S3. Free energy landscapes of binding pocket in (a) the SA system, (b) the SB system, and (c) the MR system. Parentheses showed the percentage of the variance of PC in the total variance. The colors of the low energy conformation to the high energy conformation were shown in the legend.
